# Notes on Cases in the Imperial Yeomanry Hospital at Deelfontein

**Published:** 1900-10-06

**Authors:** L. V. Cargill

**Affiliations:** Ophthalmic Surgeon to the Hospital


### Notes on Cases in the Imperial Yeomanry Hospital at Deelfontein.

By L. V. Cargill, F.R.C.S., Ophthalmic Surgeon to the Hospital.

There are now (September 10) 31 officers and 972 men to-day in hospital, 12 officers and 447 men belonging to the Imperial Yeomanry. Since the hospital started we have treated 200 officers and 3,152 men, 1,000 of whom have been Yeomanry. The medical cases are still of a mild type. One of our orderlies, who was on night duty in an enteric ward, unfortunately contracted the disease some few weeks ago, and, although the attack was of moderate severity, perforation of the bowel ensued and he died of peritonitis. He was the first orderly we have lost of enteric, although others have had it.

The enteric wards are now being emptied and thoroughly cleansed and disinfected in turn. There has been no further case of measles contracted from the one case we have had. One or two cases of patients desquamating and giving a previous history of rash and sore throat have been admitted and at once isolated.

The work in the surgical wards has been heavier during the last two or three weeks than for some months past, and six marquees have had to be transferred from the medical to the surgical side to accommodate the patients sent down. Last week, in response to an urgent telegram from Lord Methuen, Mr. Ballance journeyed north to Mafeking to see in consultation Brigadier-General Little, commanding the 3rd Cavalry Brigade, who had been seriously wounded in the right hip by a soft-nosed bullet, which had set up against a metal cigarette case that was in the General's pocket at the time. A severe lacerated wound had resulted, the entry being three inches vertically below the anterior superior iliac spine, and the exit a little below the posterior superior spine on the same side, the track being immediately adjacent to the hip joint. The General was brought down to Deelfontein in No. 5 Hospital Train, which was at Mafeking at the time, and the rest of the ninety-six patients, forty per cent. of whom were wounded, were also detrained here. General Little is now progressing favourably, and it is hoped that some movement in the hip-joint will be retained eventually. Mr. Hall-Edwards has been kept unusually busy in localising bullets and shell fragments in the X-ray department.

Last week the first amputation occurred in the hospital since its commencement in March last, and this speaks volumes for the efficiency of the modern antiseptic treatment of compound fractures and the merciful character of the Mauser bullet, a large number of compound comminuted fractures having been treated here. The case was one of severe compound fracture of both legs by the same bullet, and Mr. Ashdowne amputated the right thigh in the lower third on account of spreading osteomyelitis in the bones of the leg, which previous operations had failed to stop. The patient is now doing well, and it is hoped that the fracture in the left leg will heal.

The first circumscribed traumatic aneurism in this hospital was operated on by Mr. Parker a few days ago. The case was one of a man who was shot in the left forearm by a Mauser bullet at a range of 100 yards. The bullet entered the back of the limb one and a half inches below the tip of the olecranon process and emerged on the flexor aspect after fracturing the ulna two inches below the bend of the elbow at the anterior border of the supinator longus. The posterior wound had closed, the anterior one was still open, and on September 5th the patient suddenly lost three or four ounces of blood from it. There was a pulsatile swelling three inches in diameter on the anterior surface of the forearm just below the elbow. The radial pulse was strong; the ulna could not be felt. At the operation, after turning out the clots, an aperture was found in the ulnar artery about three-quarters of an inch from the bifurcation of the brachial. The injured vessel was tied above and below the wound and the patient is now doing well. The aneurismal cavity extended between the bones of the forearm on each side of the interosseous membrane, in which there was a hole one inch in diameter, but by far the largest part of the tumour was situated on the anterior aspect of the membrane.

We have only had a few wounds from shrapnel bullets, but their lack of penetrating power is very marked as compared with small-bore missiles. Mr. Ballance had a very interesting case of a man in whom a shrapnel bullet entered the back a quarter of an inch to the right of the middle line and on a level with the sixth dorsal spine. By means of the X rays it was localised under the sixth right rib in front, half an inch to the inner side of the mammary line, and from this position it was removed by resecting a portion of the rib, the bullet being partially imbedded in the surface of the lung.

Another shrapnel bullet has been removed from the upper end of the left femur at the level of the junction of the neck and shaft. It had entered the outer aspect of the hip and penetrated the great trochanter. One shrapnel bullet entered the outer side of the left leg on a level with the tubercle of the tibia, the shell having burst twenty yards from the patient. The bullet had just enough energy left to perforate the head of the tibia horizontally and lodge under the skin over the inner tuberosity, whence it was removed by Mr. Parker.

A most interesting case of abscess of the brain resulting from a Mauser bullet wound has been operated on in the hospital. The man was shot on April 13 near Wepener, the bullet entering the forehead one inch vertically above the inner end of the right eyebrow, and after traversing horizontally the right cerebral hemisphere it emerged in the occipital region two inches above and to the right of the external occipital protuberance, along a line drawn from this point to the right parietal eminence. He was helped off his horse and taken to hospital, and from the time of his wound had been completely paralysed in the left arm and leg. Three months later he was brought down to this hospital, with wounds long since healed, and in a very drowsy condition, with sub-normal temperature and a pulse of 54. The left arm and leg were absolutely paralysed, but not anæsthetic; the left side of the face was partially paralysed. The knee jerks were exaggerated; the urine and fæces were passed into the bed. Two days later the drowsiness had increased, and external strabismus with dilatation of the pupil had developed in the right eye. Mr. Cargill reported commencing optic neuritis on both sides, more marked on the right. Mr. Ballance then trephined over the lower part of the right Rolandic area, and after puncturing the frontal and parietal lobes without success an abscess was tapped in the occipital lobe, and from 4 to 6 drachms of thick yellow inodorous pus were evacuated. The bony aperture was enlarged posteriorly and a tube inserted. Unfortunately the patient died the next day, with a temperature of 104·4 F., never having recovered consciousness from the time of the operation. At the autopsy the abscess drained at the operation was seen to be situated in the centre o the right occipital lobe, and the cavity was about one and a half inches in diameter. There were two other smaller abscesses which had been untouched by the operation, one in the upper and one in the lower part of the right parietal lobe, near the surface of the brain. In the region of the genu of the right internal capsule a patch of white softening was found. There were no portions of bullet or clothing in the cerebral substance. In an agar tube inoculated at the operation a pure cultivation of the staphylococcus pyogenes aureus grew. Apparently the hemiplegia was due to direct destruction of the fibres of the internal capsule by the passage of the bullet, and was not due to the pressure of the abscess.

